# Sol–Gel-Derived Silica/Alumina Particles for Enhancing the Mechanical Properties of Acrylate Composite Materials

**DOI:** 10.3390/gels11080575

**Published:** 2025-07-24

**Authors:** Khaled Altwair, Vladisav Tadić, Miloš Petrović, Andrija Savić, Vesna Radojević, Radmila Jančić Heinemann, Marija M. Vuksanović

**Affiliations:** 1 Faculty of Technology and Metallurgy, University of Belgrade, 11120 Belgrade, Serbia; kaltwear@yahoo.com (K.A.); mpetrovic@tmf.bg.ac.rs (M.P.); vradojevic@tmf.bg.ac.rs (V.R.); radica@tmf.bg.ac.rs (R.J.H.); 2 Institute of Chemistry, Technology and Metallurgy—National Institute of the Republic of Serbia, University of Belgrade, 11000 Belgrade, Serbia; vladisav.tadic@ihtm.bg.ac.rs; 3 Department of Chemical Dynamics and Permanent Education, VINČA Institute of Nuclear Sciences—National Institute of the Republic of Serbia, University of Belgrade, 11351 Belgrade, Serbia; savic@vin.bg.ac.rs

**Keywords:** composites, mechanical properties, silica and alumina particles, sol–gel

## Abstract

Silica/alumina composite particles were synthesized via the sol–gel method to promote fine dispersion and homogenous mixing. Aluminum chloride hydroxide served as the alumina precursor, while amorphous silica, obtained from rice husk, was directly incorporated into the alumina sol. Following synthesis, the material was calcined at 1000 °C, yielding an α-cristobalite form of silica and corundum-phase alumina. These hybrid particles were introduced into polymer composites at reinforcement levels of 1 wt.%, 3 wt.%, and 5 wt.%. Mechanical behavior was evaluated through three-point bending tests, Shore D hardness measurements, and controlled-energy impact testing. Among the formulations, the 3 wt.% composite exhibited optimal performance, displaying the highest flexural modulus and strength, along with enhanced impact resistance. Hardness increased with rising particle content. Fractographic analysis revealed that the 3 wt.% loading produced a notably rougher fracture surface, correlating with improved energy absorption. In contrast, the 5 wt.% composite, although harder than the matrix and other composites, exhibited diminished toughness due to particle agglomeration.

## 1. Introduction

Poly (methyl methacrylate) (PMMA) is widely used across biomedical and structural applications due to its optical transparency, tunable polymerization, mechanical resilience, and biocompatibility with human tissue [1,2,3]. The adoption of PMMA has significantly advanced dental materials, and the development of blended acrylic systems has broadened its use in restorative dentistry [2,4,5]. Moreover, PMMA’s compatibility with bone tissue enables its use in orthopedic bone cements [6,7,8]. However, its intrinsic brittleness and limited toughness remain major challenges, especially in load-bearing applications [9,10]. PMMA was used as the base for shielding composites with bismuth nanoparticles [11,12]. With different additions, PMMA can be the base for different functional composites, including those with antimicrobial properties [13,14], but also for radiative cooling compositions [15], especially those containing two oxide reinforcements.

However, its intrinsic brittleness and limited toughness remain major challenges, especially in load-bearing applications. Silica is commonly used both as a standalone material and as a reinforcement in dental resins [16] for denture base materials [17,18], ballistic materials [19,20], and various functional materials [21]. Silica can be produced in several ways, ranging from standard sources using sol–gel and flame synthesis to CVD methods using various precursors. Very finely dispersed silica is naturally produced by plants such as rice [22] and is especially present in rice husks, which constitute agricultural waste [23]. Bio-based silica offers advantages in sustainability while exhibiting favorable morphology and purity suitable for nanocomposite applications [24].

Alumina as reinforcement in acrylic polymers has several functional benefits. It not only improves the mechanical properties [25] of the composite and the modulus and strength of the material but can also influence the toughness of the composite, and this is very important for this type of material [26]. Ferrous-doped alumina particles, for example, can reduce the residual monomer content in the acrylate matrix [27]. The added alumina particles also influence the surface properties of the polymer matrix composite. The added alumina particles can improve the adhesion properties of the acrylic polymer to the metal substrate. Aluminum has several hydroxides and oxides, and depending on the heat treatment and the method of preparation, it can have a list of possible applications.

Multiphase oxide materials—particularly silica/alumina hybrids—have long been explored for their phase stability and thermal resilience [28,29]. In this study, we synthesized such hybrids via the sol–gel technique, using aluminum chloride hydroxide and bio-based silica from rice husks. The use of rice-derived silica aligns with sustainability goals and offers favorable morphology at the nanoscale, even after calcination [30,31].

Although high-temperature processing at 1000 °C removes surface hydroxyl groups, particularly from the alumina phase, the resulting structure provides enhanced mechanical rigidity and thermal stability to the reinforcement. The retained fine dimensions of silica particles can contribute to strain dissipation, while the dense alumina phase ensures load-bearing reinforcement [32]. Together, this dual-phase filler is hypothesized to improve the mechanical integrity of PMMA composites not through chemical bonding but by optimized dispersion, phase contrast toughening, and crack deflection mechanisms—factors increasingly relevant in structural polymer applications. Selected amounts of reinforcement particles were based on literature coverage [33] as well as our previous experience with similar particles [18].

This approach reflects a strategy shift from interfacial reactivity to morphological and mechanical synergy, offering an alternative path for property enhancement in high-performance, biocompatible composites [34].

## 2. Results and Discussion

### 2.1. Morphological Characterization of Particles

**Figure 1:**
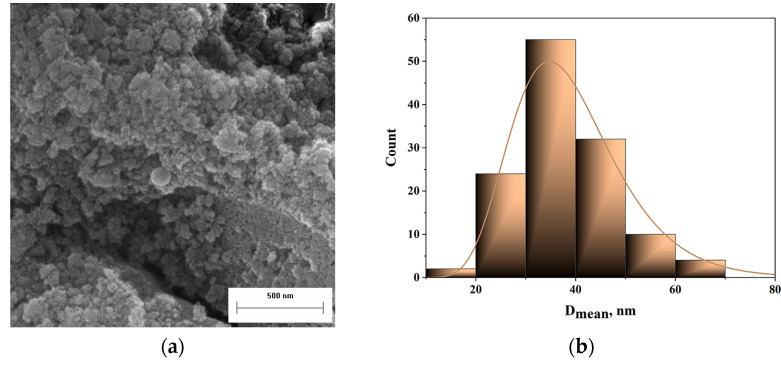
(**a**) The FESEM image of a synthesized mixture of silica/alumina particles and (**b**) their corresponding particle size distribution.

The morphological characteristics of the synthesized particles were examined using field emission scanning electron microscopy (FESEM). Although particle agglomeration was observed (Figure 1a), individual particles could still be discerned. Image analysis was employed to determine the particle size distribution, revealing that the primary particle diameter lies within the nanometer range (Figure 1b). While the micrograph clearly displays the particle morphology, it does not allow for differentiation between silica and alumina phases. However, the sol–gel synthesis route appears to facilitate a high degree of mixing between the two components. To confirm the elemental distribution and mixing uniformity, energy-dispersive X-ray spectroscopy (EDS) was utilized.

**Figure 2:**
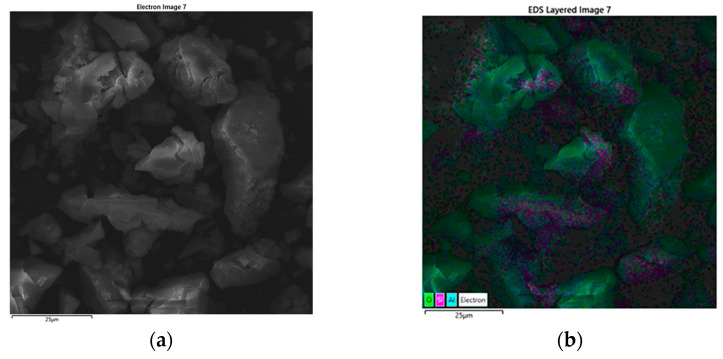

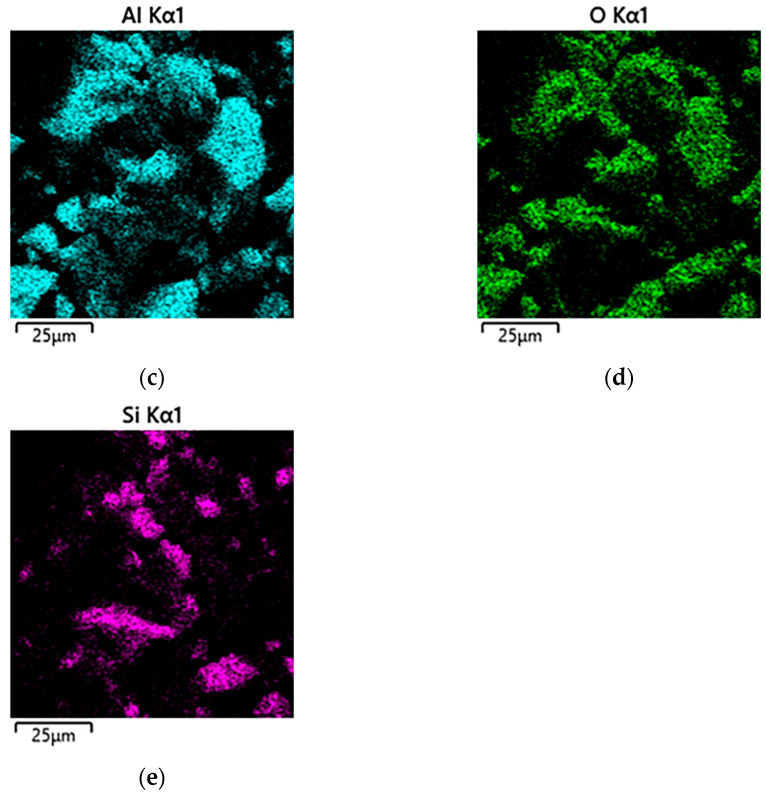
EDS mapping of silica/alumina particles from the sol–gel method: (**a**) SSEM images, (**b**) EDS of SEM image, (**c**) Al, (**d**) O, and (**e**) Si.

**Figure 3:**
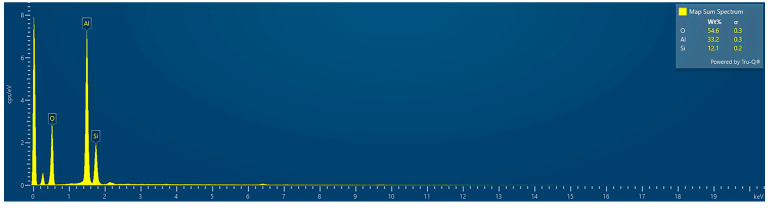
EDS spectrum of silica/alumina particles from the sol–gel process.

Energy-dispersive X-ray spectroscopy (EDS) confirms that aluminum, silicon, and oxygen are the predominant elements (Al—33.24 wt.%, Si—12.13 wt.%, and O—54.63 wt.%) within the synthesized material, indicating the successful formation of a mixed-oxide system. Elemental mapping (Figure 2b–e) reveals a high degree of spatial integration between the components, supporting the existence of a well-mixed, dual-phase structure. The silica component originates as fine particles, while the alumina phase forms in situ from a hydroxide precursor that initially coats the silica during the sol–gel process. Upon calcination at elevated temperatures, this hydroxide transforms into thermodynamically stable α-alumina, encapsulating or interspersing around the silica domains. The resulting morphology—characterized by concentrated silica cores surrounded by an alumina-rich matrix—suggests a hierarchical architecture that may influence mechanical performance through particle–matrix interactions, load transfer efficiency, and energy dissipation mechanisms. The EDS spectrum of silica/alumina particles from the sol–gel process is shown in Figure 3.

### 2.2. Structural Characterization of Synthesized Particles and Composites

**Figure 4:**
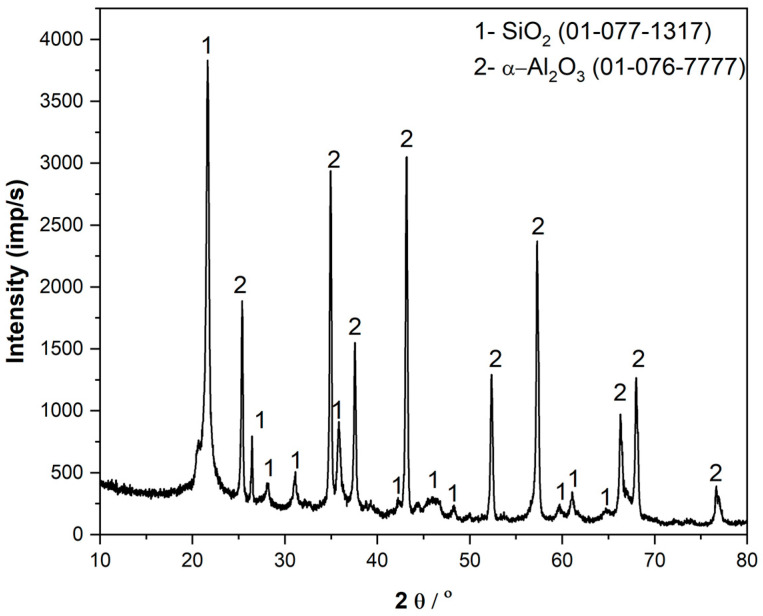
XRD pattern of SiO_2_/Al_2_O_3_ particles from the sol–gel process.

The crystallographic structure of the material was analyzed using X-ray diffraction (XRD), as shown in Figure 4. The dominant phase identified was α-alumina (corundum), the thermodynamically stable form of alumina, produced from the precursor obtained via sol–gel synthesis. The sharp, well-defined peaks in the XRD pattern indicate that the sol–gel process effectively promotes the formation of α-alumina even at 1000 °C.

The silica component, derived from plant-based material, initially exhibited a predominantly amorphous structure following calcination at 800 °C [35,36]. Subsequent heat treatment at 1000 °C led to the crystallization of the β-cristobalite phase [37,38]. In the specimen, the α-cristobalite was determined. This structure emerges through an intermediate formation from β-cristobalite obtained during calcination, which, upon cooling below approximately 270 °C, transforms into metastable α-cristobalite. This phase demonstrates promising potential for use in composite reinforcement due to its negative Poisson’s ratio [39], which facilitates stress redistribution under load [39,40]. As a result, the composite material exhibits improved modulus and strength, as observed in epoxy resin systems [41].

**Figure 5:**
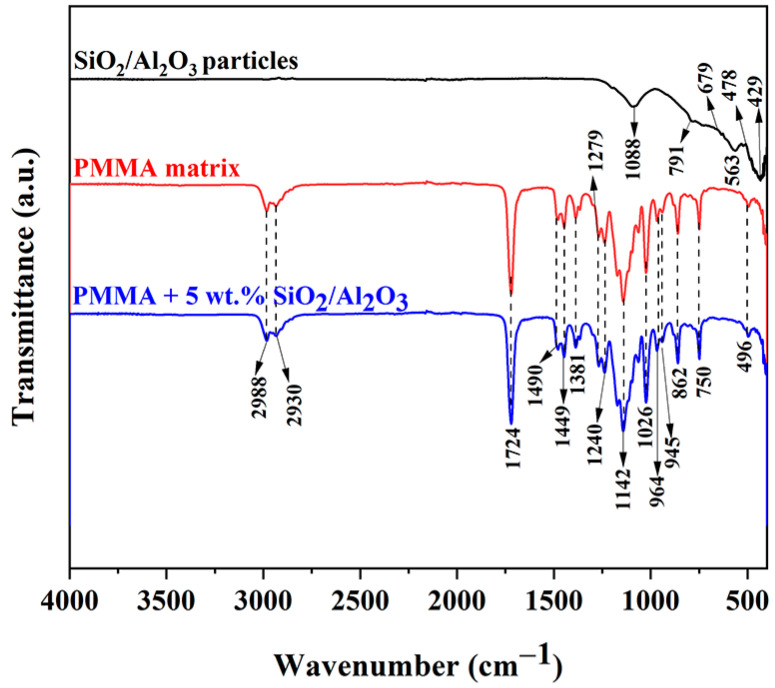
FTIR spectra of SiO_2_/Al_2_O_3_ particles, the PMMA matrix, and PMMA + 5 wt.% of SiO_2_/Al_2_O_3_ particles.

The FTIR spectrum (Figure 5) clearly exhibits the characteristic peaks of silica particles at 1088 cm^−1^, 791 cm^−1^, and 563 cm^−1^. The absorption band near 1088 cm^−1^ is attributed to the antisymmetric stretching vibrations of the Si-O-Si bonds, while the peak at 791 cm^−1^ corresponds to their symmetric stretching vibrations. Additionally, the bending vibrations of the Si-O-Si linkage are responsible for the absorption observed at 563 cm^−1^ [42]. In the lower frequency range (1000–400 cm^−1^), the infrared absorption is predominantly associated with Al-O bond vibrations [43]. A broad, shallow hump appearing in this higher frequency range, without any distinct peak, suggests a minimal presence of physically adsorbed water molecules on the surface. A pronounced broad peak detected at 679 cm^−1^ is attributed to the stretching vibrations of the Al-O bonds [44], providing strong evidence for the formation of alumina. Additionally, a smaller peak observed at 429 cm^−1^ is mainly associated with the octahedral coordination environment of AlO_6_ units [45]. The infrared (IR) spectrum of polymethyl methacrylate (PMMA) reveals several characteristic absorption bands that reflect its molecular structure. A strong peak around 1724 cm^−1^ corresponds to the stretching vibration of the carbonyl (C=O) group within the ester functionality. Another notable absorption band appears near 1449 cm^−1^, which is associated with the stretching vibrations of the carbon–oxygen (C-O) bonds. In the higher wavenumber region, two distinct bands at 2988 cm^−1^ and 2930 cm^−1^ are observed, both of which are linked to the stretching vibrations of the C-H bonds from methyl (CH_3_) groups attached to the polymer chain. These features are typical of aliphatic C-H stretches found in organic polymers. The stretching vibration of the ester C-O bond produces a pronounced absorption band at 1142 cm^−1^. Meanwhile, the vibrations corresponding to carbon–carbon (C-C) bond stretching are identified at 964 cm^−1^ and 750 cm^−1^, indicating structural details of the polymer backbone. Altogether, these spectral features are consistent with the known chemical structure of PMMA [46].

### 2.3. Mechanical Properties Results

The mechanical behavior of the material was investigated using the three-point bending method in compliance with [47] Rectangular bar-shaped specimens measuring 60 × 10 × 2 mm were tested over a support span of 30 mm, with each condition assessed in triplicate. Tests were conducted at temperatures of 20 °C with a cross-head speed of 1 mm/min. The number of specimens conforms to standard [48]. If specimens give stable results within the group prepared under the same conditions, then three specimens can be enough to draw results.

The unreinforced matrix exhibited a lower flexural modulus relative to the composite but demonstrated a higher capacity for deformation. The composite containing 1 wt.% reinforcement displayed mechanical behavior closely resembling the matrix, albeit with reduced deformational capacity. In contrast, the addition of 3 wt.% reinforcement significantly enhanced both the modulus and flexural strength by 9% compared to the matrix. However, increasing the reinforcement content to 5 wt.% did not yield further improvement; while the modulus was improved by 18% compared to the matrix, a reduction in strength was observed (Table 1).

Standard deviations remain low for all tested groups of specimens. This confirms the standardized procedure for specimen preparation and even the distribution of reinforcements, resulting in a homogeneous specimen. To prove this, the analysis of variance was performed with the experimental data, and the obtained value of *p* was 0.012, which is lower than 0.05, thus indicating that one group can be identified as stronger than the others. The group with 3 wt.% reinforcements differ significantly in values for strength from other groups, with *p* ≈ 0.048 in comparison with the matrix, *p* ≈ 0.015 in comparison with specimens with 1 wt.% reinforcement, and a *p*-value of *p* ≈ 0.043 in comparison with specimens with 5 wt.% reinforcement. The calculated *p*-value for modulus measurements is 0.037, and the group with 5 wt.% reinforcement shows significant improvement in modulus value. If groups with 5 wt.% and 3 wt.% reinforcements are compared, then the *p*-value is 0.049. The difference between those two specimens is marginally significant. Taking all of the statistical analyses into consideration, the specimen with 3 wt.% reinforcement can be regarded as the best performing among all studied groups in flexural testing.

**Figure 6:**
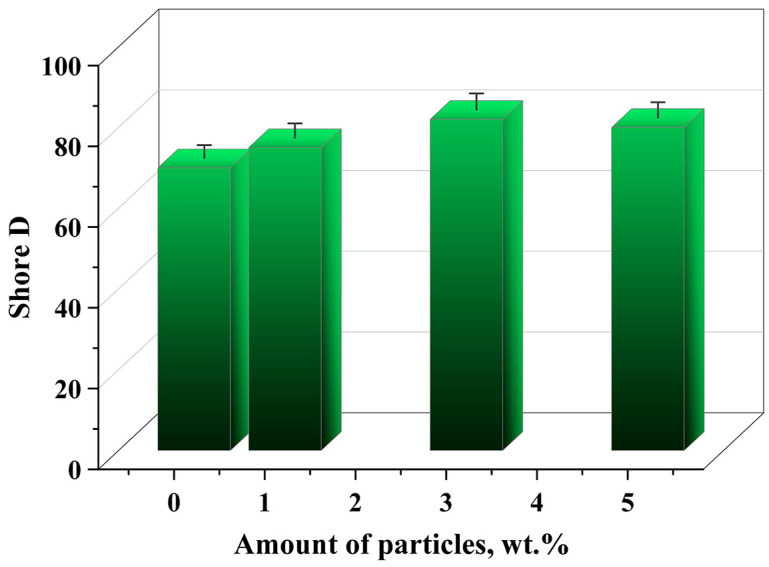
Hardness of composite materials.

The surface characteristics of the prepared composite were examined by measuring the hardness using the Shore D method and the contact angle with distilled water. The hardness of the composite increases as the content of particles is increased, and it can be concluded that the presence of particles increases the resistance of the material surface to indentation (Figure 6). Shore D is a robust method used for hard plastics, and the indenter considers the average surface under the indenter, so the increase in particle content makes the surface’s resistance to indentation stronger [49]. Increased filler content raises hardness due to restricted polymer chain mobility, reduced surface compliance, and reactions between the surface of the filler and the matrix. The increase up to 3 wt.% fillers is in accordance with the material’s optimal strengths.

**Figure 7:**
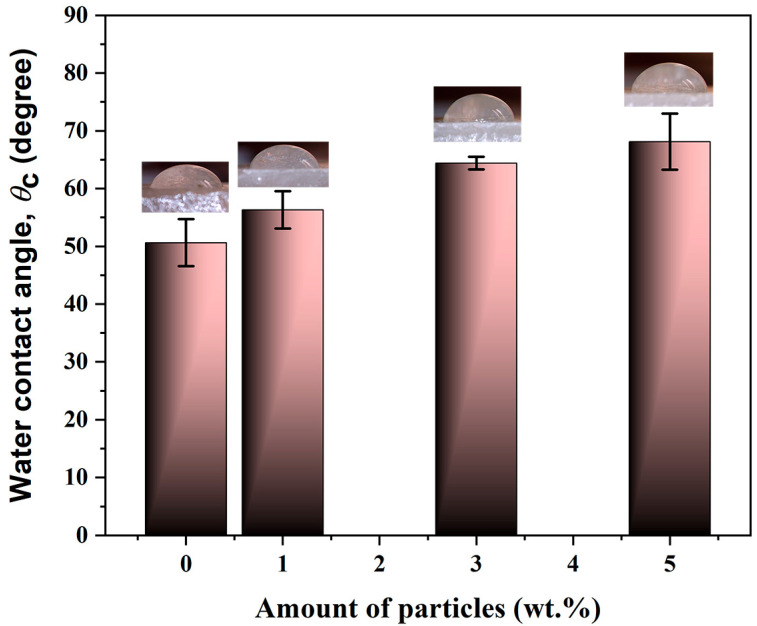
The values of static contact angles measured on the surface of PMMA and the SiO_2_/Al_2_O_3_ composite.

The surface energy was compared to distilled water by dropping a 10 µL drop on the surface and measuring the contact angle using a microscope with a magnification of 10×. The addition of particles into the composition of the material increased the contact angle, proving that the material became more hydrophobic with the addition of particles (Figure 7). This characteristic could facilitate the cleaning of the surface as the water interferes less with the surface of the material, thus containing more particles in the composition.

**Figure 8:**
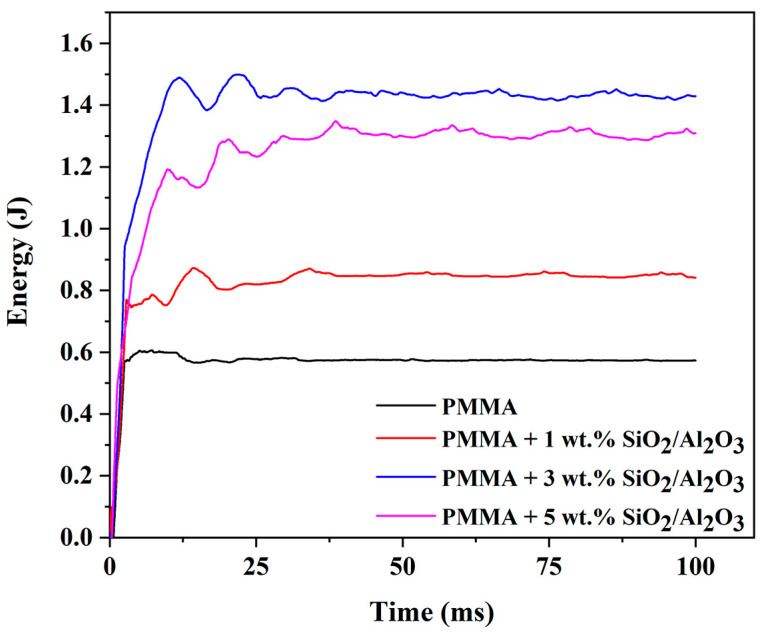
Impact testing of the PMMA matrix and PMMA composites with SiO_2_/Al_2_O_3_ particles.

Controlled energy impact testing was conducted to evaluate the material’s behavior under rapid, high-stress loading conditions, thereby assessing its toughness in severe scenarios. The unreinforced matrix exhibited limited impact resistance. However, the incorporation of mixed-oxide particles markedly improved toughness (Figure 8).

The introduction of 1 wt.% reinforcement enhanced the energy absorption capacity relative to the matrix alone. A further increase to 3 wt.% resulted in a substantial improvement—effectively doubling the energy required to fracture the specimen—highlighting the synergistic effect of silica and alumina particles in impeding crack propagation. While the composite containing 5 wt.% reinforcement showed improved toughness compared to the 1 wt.% formulation, it demonstrated lower impact resistance than its 3 wt.% counterpart. Nevertheless, the 5 wt.% composite exhibited performance characteristics well suited to the intended application.

**Figure 9:**
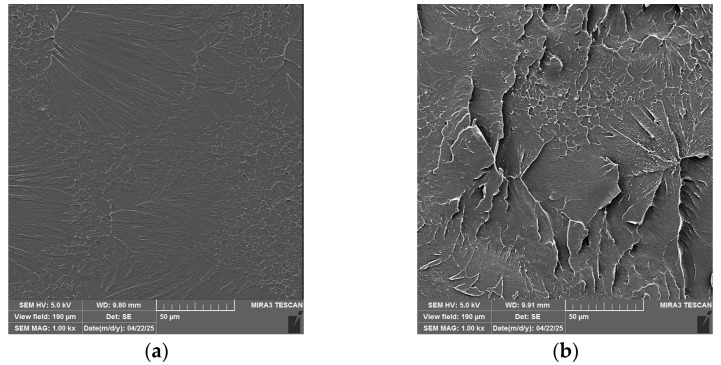

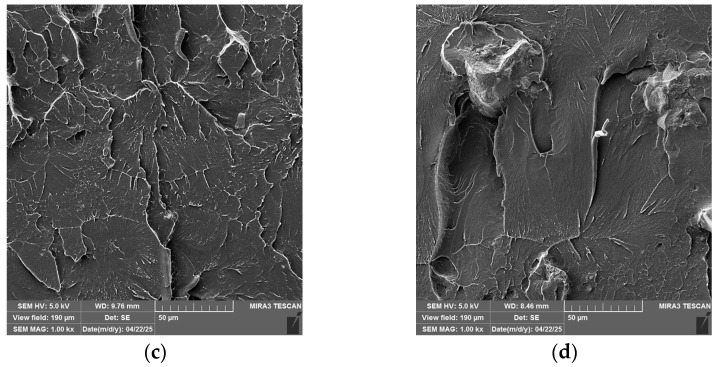
SEM images of composite materials: (**a**) PMMA, (**b**) 1 wt.% SiO_2_/Al_2_O_3_, (**c**) 3 wt.% SiO_2_/Al_2_O_3,_ and (**d**) 5 wt.% SiO_2_/Al_2_O_3_.

Fractographic analysis was conducted using field-emission scanning electron microscopy (FESEM), with representative images presented in Figure 9. The fracture surface of the unreinforced polymer matrix (Figure 9a) exhibited a smooth morphology with minimal surface features, indicative of brittle fracture behavior typical of glassy resins. The inclusion of reinforcing particles progressively increased surface roughness, as observed with 1 wt.% and 3 wt.% loadings, which produced increasingly coarse fracture morphologies.

This trend corresponds to the enhanced energy absorption observed during both tensile and impact testing, particularly up to 3 wt.% reinforcement. The specimen containing 5 wt.% particles also displayed a roughened fracture surface, suggesting higher energy dissipation compared to the neat resin. However, the presence of observable particle agglomerates in this formulation may have contributed to the reduced toughness relative to the 3 wt.% composite.

**Figure 10:**
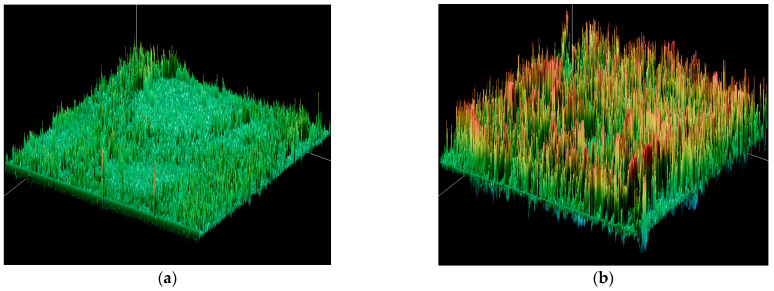

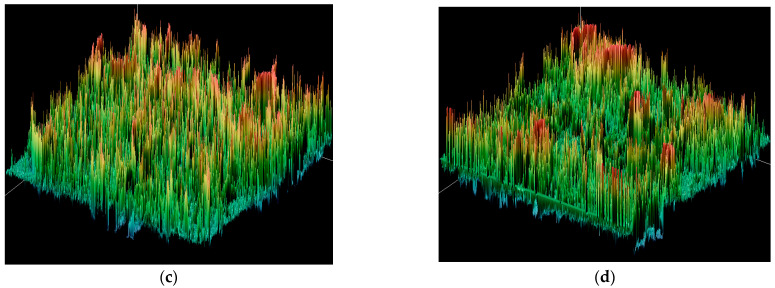
Surface plot of SEM images of the composites: (**a**) PMMA, (**b**) 1 wt.% particles, (**c**) 3 wt.% particles, and (**d**) 5 wt.% particles.

**Figure 11:**
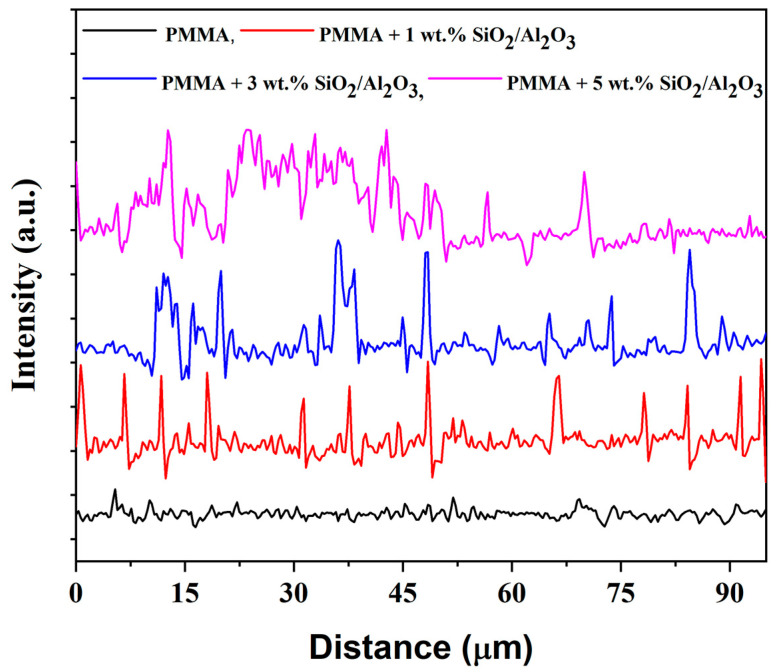
Line profiles of the surface of PMMA and SiO_2_/Al_2_O_3_ composites.

In order to quantify the observed trends in fracture surface images, some quantification techniques were used. Image Pro Plus 6.0 [50] image analysis software was used to quantify the observations of the fractographic surface. The program uses gray-level analysis to reconstruct the observed image surface, and it revealed differences that could be observed in a more visible way. The coloring of the higher parts of the specimen appears as red in images, and they represent the parts where the visible roughness in the image is present, as can be seen in Figure 10. To make this observation more visible, we performed line profile analysis, which marks the roughness of the sample, as presented in Figure 11. The matrix shows a relatively fine surface, and the observed irregularities are relatively low. This suggests that the crack propagation went through the material without any opposition. When only 1 wt.% particles are added, the roughness appears on the surface, resulting from the small presence of particles that tend to redirect the crack propagation when the material is subjected to impact. The specimen with 3 wt.% particles had a slightly rougher surface, and the profile image suggests that the propagation of cracks faces more difficulties; the particles prolonged the crack propagation path and the result was the best-performing specimen in impact testing. The specimen with 5 wt.% particles did not profit from all the particles that were added to the specimen, as due to the elevated concentration, particles were prone to agglomeration. The agglomerate was seen in the cross section after testing, and the main roughness was around this agglomeration, while the rest of the material had a lower level of observed roughness in the rest of the surface, and this can also be illustrated in line profile analysis. The agglomerations also diminish the energy absorption during the impact and do not redirect the crack propagation in the same way [51].

Surface roughness analysis of SEM line profiles (Table 2) revealed distinct topographical differences across composite formulations [52,53]. The 3 wt.% SiO_2_/Al_2_O_3_ sample demonstrated elevated roughness parameters (Ra ≈ 1700; Rq ≈ 2100; Rz ≈ 8800) with high peak density and moderately negative skewness, indicating a valley-rich profile conducive to crack blunting and mechanical interlocking. In contrast, the 5 wt.% composite exhibited the highest roughness (Ra ≈ 1920; Rz ≈ 9600) but showed slightly positive skewness, suggesting more peak-dominated features that may hinder toughness despite improved stiffness. The 1 wt.% formulation displayed minimal relief and lower roughness metrics (Ra ≈ 1280; Rz ≈ 6550), correlating with reduced flexural performance as seen in Table 2. These findings align with mechanical data, where the 3 wt.% group achieved the highest flexural strength and strain at break, indicating that surface complexity optimized at moderate filler loading enhances energy dissipation during bending and reinforces filler-matrix synergy. This highlights 3 wt.% as the formulation with the most favorable balance of modulus, strength, and interfacial toughness. More profound conclusions could be obtained once the specimen is able to undergo thermomechanical analysis, which could provide an in-depth analysis of particle–matrix interaction.

The results align with previous literature trends, though direct comparisons are limited by differing methods. In another study, unmodified alumina particles—spherical, whiskers, and bimodal (via electrospinning)—were added to a PMMA denture base. Bimodal particles yielded the best outcome, improving the modulus by 2.3 times. The results obtained for specimens with different alumina modifications and tested in a similar way as in this study have comparable results for strength and modulus [54]. It is possible to influence crack development with modified graphene particles [55].

Silica nanoparticles can influence the storage modulus in a PMMA composite, lowering it under the glass transition temperature and obstructing the movement of polymer chains at higher temperatures. This process is proportional to the content of the silica particles [56]. The storage modulus at room temperature was not considerably influenced by the addition of particles. The use of modified silica particles of up to 1 wt.% improves the storage modulus, as well as disrupting the movement of polymer chains, as shown by using dynamic mechanical analysis [57]. Silica obtained from rice husks can have a fine nanocrystalline structure and be of a submicrometer size. When added to the PMMA denture base material, it improves the polymer’s hardness [58]. In the present publication, the structure derived from rice husk silica transforms into an α-cristobalite structure, and alumina transforms to a corundum structure. Therefore, their properties improve up to 3 wt.% of added particles in terms of modulus and strength, but further addition of particles makes them more susceptible to agglomeration. The interaction between the particles and the matrix is limited due to the crystal structure, limiting the improvement of toughness, contrary to results obtained when the particles were calcinated at lower temperatures. Combined alumina zirconia particles were used in a different ratio as reinforcement of the PMMA matrix for the denture base at an amount of 5 wt.%, and the specimens were tested through two-point bending and tensile testing. The content of particles was higher than in the present study. We obtained optimal results and the materials were comparable but a bit higher in value for strength and modulus [59].

## 3. Conclusions

Combined particles of silica, mainly with an α-cristobalite structure, and alumina, mainly with a corundum structure, were prepared using the sol–gel technique, and this was observed using an XRD pattern study. The calcination temperature was 1000 °C, which was enough to produce particles that enable noticeable improvement of the mechanical properties of the PMMA matrix composite. The composite was prepared with 1 wt.%, 3 wt.%, and 5 wt.% particles, and it was tested using a three-point bending test, a hardness test, and finally by being exposed to a controlled energy impact test. The composite with 5 wt.% reinforcement presented 18% improvement, the composite with 3 wt.% reinforcement improved the modulus by 9%, and the 1 wt.% addition of particles did not influence the flexural modulus compared to the matrix material. The flexural strengths showed 10% improvement in the specimens with 3 wt.% reinforcement, and the specimens with 5 wt.% reinforcement did not show improved flexural properties at all. Toughness was significantly improved in specimens with 3 wt.% and 5 wt.% reinforcements, showing double the amount of energy needed to break them in controlled energy impact tests. The best performing specimen was the one containing 3 wt.% reinforcement; it produced improved modulus, strength, and toughness compared to the other specimens. Addition of 5 wt.% made the dispersion of particles complicated, and agglomerates formed. Studies of the fracture surface explain those findings as the fracture surface becomes rougher with the addition of particles into the composite. The wettability of the composite is lower compared to the pure matrix, as can be seen from the contact with a water drop. The results show that adding 3 wt.% SiO_2_/Al_2_O_3_ nanoparticles gives the best balance of fracture surface roughness and mechanical properties. This composition achieved optimal topography for strong particle–matrix bonding, resulting in the highest flexural strength and toughness. Higher filler content (5 wt.%) increased fracture surface roughness further but reduced toughness, while lower content (1 wt.%) led to weaker properties. Overall, 3 wt.% is optimal for improved composite performance.

## 4. Materials and Methods

The matrix was prepared using the standard dental polymer set, which is applied in dentistry for dentures and orthodontics. ORTO poly prepared by Polydent, Renče-Vogrsko, Slovenia, was used for cold polymerization. Particles were prepared using the sol–gel method. The precursor for the alumina component was Al_2_(OH)_5_Cl 2.5 H_2_O; it was purchased as Locron from Degussa company, Berlin, Germany. Silica was used in the form of fine particles obtained from rice husk (Levidiagro, Kočani, North Macedonia). The water used was demineralized water.

Silica particles were derived from rice husk as a sustainable precursor. The rice husk was thoroughly washed with water, oven-dried, and treated with 10% sulfuric acid at 80 °C for 3 h. Following acid treatment, the material was rinsed with distilled water until a neutral pH was achieved and subsequently dried at 50 °C. The resulting black residue, containing residual organic matter, was subjected to open-flame combustion and then heat-treated at 800 °C for 4 h in an oxidative atmosphere. The final white silica-rich powder was used in subsequent synthesis steps [58].

To ensure effective mixing of components, the obtained silica was combined with aluminum chloride hydroxide sol. The mass ratio was designed to yield a calcinated product with a target molar ratio of Al_2_O_3_:SiO_2_ = 3:2. The mixture was allowed to undergo gelation, followed by drying and grinding. The resulting gel-derived composite was then calcined at 1000 °C for 3 h to produce the final oxide blend.

Composites were prepared using synthesized particles and PMMA. The precursor consists of the liquid component and a powder. Building on prior research with individual oxide reinforcements, we selected 1 wt.%, 3 wt.%, and 5 wt.% loadings for the hybrid system, as, within this range, optimal improvements in mechanical performance without compromising processability were achieved—balancing functionality with material constraints typical in dental-grade acrylics. The particles were dispersed in the liquid in an ultrasonic bath for 15 min and then mixed with the powder. As the powder contains the initiator, polymerization took place within 1 h. The concentration of particles was chosen to be 1 wt.%, 3 wt.%, and 5 wt.%, and composites were compared to the matrix material. In the following text, the sample designations are PMMA for the matrix and PMMA + 1 wt.% SiO_2_/Al_2_O_3_, PMMA + 3 wt.% SiO_2_/Al_2_O_3_, and PMMA + 5 wt.% SiO_2_/Al_2_O_3_ for the composites.

The crystal structure underwent XRD testing. The Ultima IV Rigaku (Tokyo, Japan) diffractometer employs the Bragg–Brentano geometry and uses CuKα radiation (λ = 1.5418 A) with a generator voltage of 40.0 kV and a current of 40.0 mA. The crystalline phase of silica/alumina particles was determined using continuous scan mode with a step size of 0.02° and a scan rate of 10°/min, covering a range of 10–80° 2θ. The possible interactions between the reinforcement and matrix were determined using the FTIR technique. FTIR spectroscopy was performed using a Nicolet 6700 spectrometer (Thermo Scientific, Tsuen Wan New Town, China) in attenuated total reflection mode at 4 cm^−1^. The spectra were acquired in the wavelength range of 4000 to 400 cm^−1^. The morphology of the particles was visualized using FESEM and the EDS technique to monitor the mixing degree of produced reinforcement. The dispersion of particles in the composite was also monitored using FESEM (Mira3 Tescan, Oxford, UK at 3 kV). Energy-dispersive spectroscopy (EDS) analysis was performed via an INCAx-act LN2-free Analytical Silicon Drift Detector (Oxford Instruments, Oxford, UK), in conjunction with the PentaFET ^®^ Precision and Aztec 4.3 software suite (Oxford Instruments, Oxford, UK), linked to the TESCAN Mira3 XMU. The specimen holder was made of steel, and the conductive carbon-based band was placed on it to hold the specimens. Mechanical testing of the specimens was performed using the three-point bending test and tension test with a Texture Analyzer EZ Test LX (Shimadzu, Kyoto, Japan). The 3-point bending test was performed on specimens of 60 mm × 10 mm × 2 mm, and the specimens for tension testing had 5 cm lengths, 5 mm widths, and 2 mm thicknesses. The employed controlled energy impact testing machine was a Hydroshot HITS-P10 Shimadzu, Kyoto, Japan. The toughness was monitored using the controlled energy impact test on specimens of 60 mm × 60 mm × 2 mm. Hardness was determined with a Shore D durometer (Model HBD 100-0, Sauter, Balingen, Germany). ShoreD is the method for rapidly measuring the hardness of polymer composites using the indentation needle with a calibrated weight load. Wettability was measured using an optical USB microscope (5.0 MP PRO Delta Optical Smart, Warsaw, Poland) with 40× magnification and image analysis software, ImageProPlus 6.0 (Media Cybernetics, Rockville, MD, USA), for contact angle measurement.

## Figures and Tables

**Table 1:** Flexural properties of specimens.

Specimen	Flexural Modulus, E_f_ (GPa)	Flexural Strength, σ_f_ (MPa)	Flexural Strain at Break, ε_fM_ (%)
PMMA matrix only	1.62 ± 0.081	59.07 ± 2.95	6.75
PMMA + 1 wt.% SiO_2_/Al_2_O_3_	1.64 ± 0.085	56.91 ± 2.67	4.74
PMMA + 3 wt.% SiO_2_/Al_2_O_3_	1.76 ± 0.089	64.42 ± 3.16	5.29
PMMA + 5 wt.% SiO_2_/Al_2_O_3_	1.92 ± 0.092	57.26 ± 2.86	4.00

**Table 2:** Summary of data obtained from line profile analysis. Calculations were made according to the SEM images shown in Figure 9.

Composite	Ra (Units)	Rq (Units)	Rz (Units)	Skewness	Flexural Strength (MPa)	Maximum Energy in Impact Test J
PMMA matrix only	~1100	~1350	~5200	Negative	59.07	0.6
PMMA + 1 wt.% SiO_2_/Al_2_O_3_	~1280	~1580	~6550	Negative	56.91	0.8
PMMA + 3 wt.% SiO_2_/Al_2_O_3_	~1700	~2100	~8800	Negative	**64.42**	1.4
PMMA + 5 wt.% SiO_2_/Al_2_O_3_	**~1920**	**~2350**	**~9600**	Positive	57.26	**1.3**

## Data Availability

The data presented in this study are available from the corresponding author or co-authors upon request. The data are not publicly available.

## Abbreviations

The following abbreviations are used in this manuscript:

PMMA	Poly (methyl methacrylate)
FTIR	Fourier Transform Infrared Spectroscopy
SEM	Scanning electron microscope
EDS	Energy-Dispersive Spectroscopy
